# The involvement of patient organisations in rare disease research: a mixed methods study in Australia

**DOI:** 10.1186/s13023-016-0382-6

**Published:** 2016-01-12

**Authors:** Deirdre Pinto, Dominique Martin, Richard Chenhall

**Affiliations:** Centre for Health Equity, School of Population and Global Health, The University of Melbourne, Victoria, 3010 Australia

**Keywords:** Rare diseases, Patient organisations, Research, Australia, Research policy, Patient and public involvement, Consumer involvement

## Abstract

**Background:**

We report here selected findings from a mixed-methods study investigating the role of Australian rare disease patient organisations (RDPOs) in research. Despite there being many examples of RDPOs that have initiated and supported significant scientific advances, there is little information – and none at all in Australia – about RDPOs generally, and their research-related goals, activities, and experiences. This information is a pre-requisite for understanding what RDPOs bring to research and how their involvement could be strengthened.

**Methods:**

We reviewed 112 RDPO websites, conducted an online survey completed by 61 organisational leaders, and interviewed ten leaders and two key informants. Quantitative and qualitative data were analysed using basic descriptive statistics and content analysis, respectively.

**Results:**

Although most are small volunteer-based groups, more than 90 % of the surveyed RDPOs had a goal to promote or support research on the diseases affecting their members. Nearly all (95 %) had undertaken at least one research-related activity – such as providing funding or other support to researchers – in the previous five years. However, RDPO leaders reported considerable challenges in meeting their research goals. Difficulties most frequently identified were insufficient RDPO resources, and a perceived lack of researchers interested in studying their diseases. Other concerns included inadequate RDPO expertise in governing research “investments”, and difficulty engaging researchers in the organisation’s knowledge and ideas. We discuss these perceived challenges in the light of two systemic issues: the proliferation of and lack of collaboration between RDPOs, and the lack of specific governmental policies and resources supporting rare disease research and patient advocacy in Australia.

**Conclusion:**

This study provides unique information about the experiences of RDPOs generally, rather than experiences retrospectively reported by RDPOs associated with successful research. We describe RDPOs’ valuable contributions to research, while also providing insights into the difficulties for small organisations trying to promote research. The study is relevant internationally because of what it tells us about RDPOs; however, we draw attention to specific opportunities in Australia to support RDPOs’ involvement in research, for the benefit of current and future generations affected by rare diseases.

## Background

Each of the 6000 to 7000 rare diseases^1^ [1] affects relatively few people, but collectively rare diseases affect an estimated six to eight per cent of the population [2]. Based on this estimate and Australia’s 23.7 million population at the end of 2014 [3], it is likely that at least 1.4 million Australians have a rare disease. Because rare diseases are usually life-threatening or chronically disabling, and most have no specific or effective treatments [4, 5], they impact severely on affected individuals and their families [6]. However, the low prevalence of individual rare diseases means they have historically received little attention from government or industry research funders [4, 5, 7, 8].

In the absence of resources from other sources, support from rare disease patient organisations (RDPOs) – defined here as non-profit community groups representing people affected by specific rare diseases – may be crucial to the initiation and progress of biomedical research [9–11]. Like patient organisations for more common diseases, RDPOs emerged in the United States (US) around the middle of the 20^th^ century [10] and have since proliferated across the Western world. While the traditional function of RDPOs is to support patients and/or their families, these organisations are believed to be playing increasingly active roles in rare disease research [12–14]. Indeed, there are now many examples of RDPOs that have initiated and/or supported significant programs of research [10, 13, 15–18], including the development of new therapies for diseases such as cystic fibrosis [19, 20], Pompe’s disease [21], Giant Axonal Neuropathy [13], and lymphangiomyomatosis [22].

Internationally, there is growing interest in strengthening RDPOs’ engagement in research [14, 23, 24]. This trend is observed in the context of greater prioritisation of rare disease research by governments concerned about the collective public health impacts of rare diseases [5, 25, 26] and pharmaceutical companies attracted to rare diseases as untapped markets [27]. RDPO-researcher collaborations are also of interest to policy-makers responsible for promoting the involvement of patients and their representatives in research [14, 28, 29], and to social scientists concerned with how patient communities influence the directions, practices and cultures of biomedical science [30, 31].

Despite the practical and theoretical significance of the topic, there is limited information about how RDPOs are involved in research. The relevant literature consists mainly of case studies and reports of individual RDPOs [9]. These accounts usually focus on organisations that have successfully influenced research agendas and outcomes, which may not be representative of RDPOs generally. While a few surveys [32–35] have examined the research-related activities of larger numbers of RDPOs, their authors provide limited information about RDPO leaders’ experiences in supporting research. Understanding these experiences – including any challenges or problems encountered by RDPOs that seek to advance research – is an important underpinning for initiatives to strengthen RDPOs’ contributions and collaborations with researchers.

### Australian context

As other authors have noted, previous studies of patient organisations’ roles in research have been conducted only in Europe and the US [30, 36]. In the absence of studies or even descriptive reports of Australian RDPOs, little is known about these organisations or their involvement in rare disease research. The need for country-specific information as a basis for policy development is highlighted by evidence that the involvement of patient organisations in research (for both rare and common diseases) is influenced by the political and social context in which the organisation is situated [30, 32, 36–38].

The environment for rare disease research and patient advocacy is very different in Australia compared with either Europe or the US. As discussed later in this article, there are no specific policies or programs supporting Australian rare disease research [38–41], with the exception of “orphan drug” legislation providing tax benefits and extended periods of market exclusivity for pharmaceutical companies that develop drugs for rare and neglected diseases [5, 42]. In contrast, Europe and the US have significant government-funded programs and initiatives targeting rare disease research [5, 25, 43–45], including a strong development program for infrastructure such as registries and biobanks that can be used in the study of any rare disease [27, 46, 47]. There are also major initiatives in both Europe and the US to strengthen RDPOs’ involvement in rare disease research and related deliberative processes [23, 28, 34, 48–50].

### Aim of our study

In this article, we report selected findings from a study investigating the research-related goals, activities and experiences of Australian RDPOs. In an effort to overcome the limitations of previous studies, such as the lack of representativeness of case studies and the restricted depth of analysis possible in surveys, our study included three different methods: an analysis of RDPO websites; an online survey of Australian RDPO leaders; and in-depth interviews with ten RDPO leaders and two key informants.

We describe the characteristics of Australian RDPOs; evaluate their research ambitions and modes of involvement in research; and discuss the challenges that Australian RDPO leaders identify with their efforts to contribute to research. We consider this novel information in the context of current Australian research policies and identify a number of specific opportunities to support RDPOs’ involvement in research and their collaborations with researchers, so as to capitalise more fully on their potential contributions.

## Methods

### Identification of eligible RDPOs

RDPOs eligible for inclusion in this study were patient organisations that focus on a disease, or closely related set of diseases, with a community prevalence of 1 in 2000 or less. We also required that organisations met the criteria for membership of the International Association of Patient Organisations [51], and therefore included only non-profit, non-government groups with a legal status (such as incorporation) in Australia. Further, the study was restricted to organisations with a website, as this was necessary to check the organisation’s eligibility for the study and collect contact details for the planned online survey. Candidate organisations were initially identified from support groups listed on the websites of two Australian rare disease alliance groups – Rare Voices Australia [52] and the Association of Genetic Support of Australasia (now Genetic Alliance Australia) [53] – and from charities listed on the Australian Charities and Not for Profits Register [54]. Further organisations were identified using a snowballing method. We identified 117 eligible patient organisations.

### Website screening and data extraction

All RDPO websites were screened during the initial identification process (August to October 2013) to confirm eligibility for the study, collect contact details, and inform the questions and code sets for the online survey.

At a later stage (January to March 2014) we analysed the websites of 112 RDPOs (the other five websites contained inadequate information or were not functioning in the analysis period). Website content was read in its entirety – usually excluding any blogs and linked documents or audio-visual material. Data on organisational characteristics, and stated research goals and activities, if any, were recorded in a spreadsheet.

### Survey

In late October and early November 2013, the leaders of three RDPOs helped to pilot test a questionnaire. On 4 December 2013, the leaders of the remaining 114 RDPOs were invited to participate in an online survey hosted by an independent survey research company.

Respondents were asked to consider all aspects of research on their diseases, from basic science studies to the development and testing of diagnostic tools, therapies and rehabilitative approaches, and research investigating the impact of the disease on patients, families or society. The questionnaire asked about RDPOs’ goals, including research goals, and research-related activities in the last five years. All respondents with a goal to support research were asked how they would ideally like to contribute to research, and what they considered the barriers to their further involvement and to the progress of research on the disease. We also collected data on organisational characteristics, such as age, number of members and staff, funding sources, and budget.

By 8 March 2014, when the survey was closed, completed surveys were received from 61 RDPOs. The final response rate of 53 % was achieved using one email reminder and one telephone reminder. Survey respondents and “non-responders”^2^ were compared according to the age of their RDPOs, geographical coverage (state-based, national etc.), disease prevalence, and whether or not research goals were indicated on the organisation’s website. No significant differences were found between survey respondents and “non-responders” on any of the variables examined.

### Interviews

Survey recipients were asked to indicate their willingness to be interviewed as part of the study. Ten RDPO leaders who agreed were selected to participate in semi-structured face-to-face interviews. Two key informants were also interviewed in order to better understand the broader context for rare disease patient organisations and rare disease research in Australia. The key informants had extensive experience in working with Australian RDPOs.

### Data analysis

Due to the small size of the survey sample, the analysis of quantitative survey data was limited to basic descriptive statistics (frequency counts and percentages, cross-tabulations, and correlations). These data were analysed using the Statistical Package for the Social Sciences. Qualitative information from answers to open-ended survey questions and interviews was subject to content analysis to identify key themes.

### Ethics

The study was approved by The University of Melbourne’s School of Population and Global Health Human Ethics Advisory Group. Survey respondents had the option to remain anonymous to the researchers, although most chose to provide identifying information on the questionnaire. To protect their confidentiality, each study participant quoted in this article is referred to as a “survey respondent” or – where interviewed – by a pseudonym.

## Results

Our report of findings from our study of Australian RDPOs begins with their basic characteristics (age; formation and governance; size and resources), followed by the results of our survey question on key organisations goals. We then consider various research-related activities conducted by RDPOs in the five years prior to the survey. Finally, we present qualitative information, from open-ended survey questions and interviews, about the challenges identified by study participants when asked about the impacts of and constraints on their involvement in research.

### Characteristics of Australian RDPOs

#### Age

**Fig. 1:**
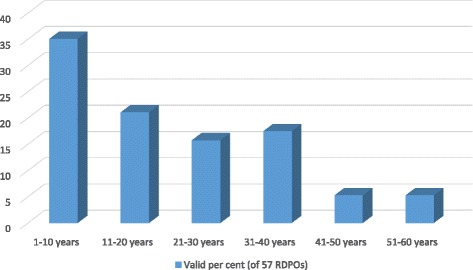
Age of Australian rare disease patient organisations. The proportion of Australian RDPOs falling into each of six age categories, based on survey responses from 57 RDPO leaders who provided information about the year in which their organisation was formed

Consistent with international trends [55], Australian patient groups focused on rare diseases emerged in the 1950s. The oldest RDPOs – for people affected by two of the more common rare diseases, muscular dystrophy and cystic fibrosis – were up to sixty years old at the time of the survey. However, most current RDPOs are much younger. More than half the surveyed RDPOs had been in existence less than 20 years, with about one third less than a decade old (Fig. 1).

RDPOs aged ten years or less at time of the survey differed from older groups in a number of respects. First, the more recently formed RDPOs were associated with lower prevalence diseases (*p* < 0.05). Second, the younger groups were smaller: they had lower 2012-13 budgets (*p* < 0.05), fewer members (*p* < 0.05) and fewer paid employees (*p* < .05). Third, the younger groups were much less likely than the older groups to raise funds directly from their members and membership fees (*p* < 0.01). Fourth, as detailed under *Organisational goals*, newer RDPOs placed a higher priority on research relative to other organisational goals (*p* < 0.05).

#### Formation and governance

More than eighty per cent of the surveyed RDPOs were founded either by patients with the disease (29 %); parents or other family members (41 %); or both patients and family members (14 %). Two (3 %) of the 59 leaders who answered this survey question said their organisation was founded by researchers or health professionals, and another seven (12 %) said their RDPO was founded by patients or families working jointly with researchers or health professionals. The survey did not provide a separate response option for patients or family members who are themselves researchers or health professionals, but this was the case for at least one RDPO.

While our survey elicited details of RDPO founders rather than current leaders, as 55 of the 61 respondents identified themselves, we were able to obtain information about 55 current leaders from organisational websites. On this basis, we estimated that approximately three quarters of RDPOs are currently led by volunteers who are personally affected by the organisation’s focus disease, either as patients or family members. The remaining RDPOs – which tend to be larger, older and devoted to higher prevalence diseases – employ professional administrators in leadership positions. These organisations had started as small volunteer groups, and over time evolved into formal organisations with professional leadership, paid staff and higher capacity to raise funds.

#### Size and resources

The surveyed RDPOs varied widely in terms of their membership numbers, budgets, and staffing; however most were of modest size. Approximately one third (32 %) had sixty or fewer members, and more than a quarter (27 %) had a total budget of $10,000 or less in 2012–13 (see Table 1). As mentioned, only a minority (an estimated 23 %)^3^ had paid leaders, and more than half (56 %) had no paid staff at all.

**Table 1:** Size and resources of surveyed Australian rare disease patient organisations

	Number of RDPOs (per cent of 61 survey respondents)	Valid per cent
*Geographical coverage*		
Specific states or territories^a^	19 (31.1)	31.7
Whole of Australia^b^	30 (49.2)	50.0
Australasian	9 (14.8)	15.0
Australian arm of international organisation	2 (3.3)	3.3
Not stated	1 (1.6)	-
*Number of members*		
60 or fewer	17 (27.9)	32.1
61 – 300	21 (34.4)	39.6
More than 300	15 (24.6)	28.3
Not stated	8 (13.1)	-
*Number of paid employees*		
No paid employees	32 (52.5)	56.1
1–2	8 (13.1)	14.0
3–5	6 (9.8)	10.5
6–15	7 (11.4)	12.3
More than 15	4 (6.6)	7.0
Not stated	4 (6.6)	-
*Total budget in 2012–13*		
$0 to $10,000	15 (24.6)	27.3
$10,000 to $50,000	9 (14.8)	16.4
$50,000 to $100,000	5 (8.2)	9.1
$100,000 to $200,000	10 (16.4)	18.2
$200,000 to $500,000	5 (8.2)	9.1
$500,000 to $1,000,000	2 (3.3)	3.6
Over $1,000,000	9 (14.8)	16.4
Don’t know/not stated	6 (9.8)	-
*Sources of funding in 2012–13* ^c^		
Membership fees	35 (57.4)	58.3
Donations and bequests	48 (78.7)	80.0
Fundraising events	46 (75.4)	76.7
Government	16 (26.2)	26.7
Industry pharma or biotech company	12 (19.7)	20.0
Philanthropic organisations	19 (31.1)	31.7
Community or business sponsors	21 (34.4)	35.0
No funding	1 (1.6)	1.7
Funded by founder	2 (3.3)	3.3
Other/don’t know	2 (3.3)	3.3

^a^Includes state-based RDPOs of RDPO federations with separate national body

^b^Includes national RDPOs with separate state organisations

^c^Totals exceed 100 % as respondents could choose more than one response. One respondent did not complete the section on organisational details

Most of the wealthier RDPOs were several decades old and dedicated to higher prevalence rare diseases. Compared with lower budget organisations, the bigger budget RDPOs were more likely to receive funding from community and business sponsors (*p* < 0.01), philanthropy groups (*p* < 0.001), and governments (*p* < 0.01). On the whole though, most RDPOs rely heavily on annual membership fees (usually between $20 and $50 per member), small donations, the occasional bequest, and “grassroots” fundraising events (such as fun runs, movie nights, charity dinners, sausage sizzles and so on). Only about a quarter of the surveyed RDPOs received government funding (Table 1). Information on RDPO websites suggested that government funding comes mainly from small grants programs for disability self-help groups or – for bigger RDPOs with professional staff – a funding agreement in which the RDPO provides allied health, clinical or disability support services. The results in Table 1 also show that twenty per cent of the surveyed RDPOs had received funding from a pharmaceutical or biotechnology company in 2012–13.

### Goals

Given RDPOs’ historical role as self-help and support groups, it is notable that 92 % of the surveyed leaders indicated a goal to “support or promote research”, and 59 % indicated a goal to advocate to authorities about research-related matters. Nevertheless, most RDPOs also have goals more traditionally associated with patient organisations (see Table 2), with 90 % aiming to provide social support and networking opportunities to members, and 97 % aiming to “provide information or education to patients and/or their families”. RDPO websites showed that nearly all leaders compile information about their RDPO’s focus disease, based on internet sources, discussions with medical experts, and/or the experiences of people affected by the disease. In addition to using their knowledge to educate RDPO members, most leaders wish to inform the general public (97 %) and health professionals (82 %) about the disease. RDPO leaders also use their knowledge in discussions with researchers and in deciding what research their organisations will fund and support.

**Table 2:** Goals of RDPOs

Goal^a^	Number of RDPOs (per cent of 61 survey respondents)^b^
To raise community awareness and knowledge of the disease	59 (96.7)
To provide information or education to patients and/or their families	59 (96.7)
To provide information or education to health professionals	50 (82.0)
To provide social support or networking opportunities for patients and/or their families	55 (90.2)
To provide services (for example, respite care), financial assistance, or other resources (for example, equipment) to patients and/or their families	25 (41.0)
To support or promote research on the disease	56 (91.8)
To advocate to government or other authorities for research funding, other research resources, or changes in regulations relating to research	36 (59.0)
To advocate to government or other authorities on other matters (for example, access to services or existing therapies)	41 (67.2)

^a^The code set for this question was based on the website review, which revealed that RDPOs’ statements of goals (or missions, priorities and so on) could be categorised according to the eight statements listed in the table

^b^Totals equal more than 100 % because respondents could choose more than answer

#### Relative priority of research and other organisational goals

Nine leaders (15 %) identified research as their “top priority” when asked about the priority of research compared to other organisational goals, and a further five (8 %) said it was “somewhat more important than other goals”. Seventeen RDPOs (28 %) had other goals that were more important than research,^4^ and the remaining thirty respondents (49 %) considered research and other goals to be equally important. Thus, almost three quarters of surveyed leaders felt that their research goals were at least as important as other goals.

Leaders’ ratings of the relative importance of research and other organisational goals were significantly negatively correlated with the age of the organisation: that is, leaders of newer groups considered research a higher priority than did leaders of older RDPOs. This result was observed even though older, more established organisations had significantly bigger budgets and were more likely to have funded research in the last five years. Many older RDPOs have broadened their original goals to include research, but our data suggest that there has also been a concurrent formation of new “research-focused” RDPOs. Based on their recent review of literature on patient organisations’ involvement in “genomic science”, Koay and Sharp predict that the trend towards the increasing formation of research-engaged patient organisations will continue and strengthen in future years [11].

While our sample size was not large enough to allow us to statistically validate “types” of RDPOs, we did observe differences between leaders according to their depth of interest in research. Based on their websites, survey comments, and (in two cases) interview data, the nine survey participants who rated research as their top priority appeared driven by a personal ambition to contribute to advances in treating diseases that had strongly affected their own lives, either as patients (three cases), parents of a living or recently deceased child (five cases) or a close friend (one case) of someone with the disease. In eight out of the nine cases, the diseases were degenerative conditions not apparent at birth: the RPDO leaders believed these diseases to be potentially curable but neglected by the research establishment. These leaders tended to be very proactive in trying to initiate new research and in developing relationships with researchers. Seven of these nine RDPOs had been established with the primary or exclusive purpose of advancing research, and some of the leaders appeared to have little interaction with other members of the patient community.

Compared with the RDPOs that gave greater priority to research, RDPOs with equal interest in research and other goals tended to be older and to have larger memberships. They had usually started as patient support groups but had become involved in research as their resources grew, scientific knowledge advanced and/or opportunities for participation presented. Unlike the leaders of highly research-focused groups, leaders with “balanced goals” wished to support research but did not necessarily regard their RDPOs as being responsible for initiating new programs of work or changing research agendas. They also tended to see their research and patient support goals as intertwined, with several commenting that research involvement brought hope to their members and strengthened ties between them.

RDPOs with the least interest in research were typically small support groups, some of which were devoted to conditions – such as spontaneous congenital malformations – for which there appear to be little likelihood of cures in the foreseeable future. The research involvement of these organisations, if any, tended to be driven by researchers (for example, responding to researchers’ request to advertise their studies on RDPO websites) or raising small amounts of money for a particular hospital or other institution with which they have a close relationship.

### Research activities

Overall, 95 % of the surveyed RDPOs had undertaken at least one research-related activity, as listed in Table 3, in the last five years.

**Table 3:** Research-related activities undertaken by RDPOs in the last five years

Activity	Number of RDPOs (percent of 61 survey respondents)^a^
Provided funding to researchers, research bodies or a specific research fund/foundation separate to the RDPO	36 (59.0)
Collaborated with or provided non-financial support to researchers or research bodies	47 (77.0)
Established or maintained the organisation’s own patient registry (i.e. a data set containing clinical information about patients) or biobank (collection of biological samples)	7 (11.5)
Contributed to an external patient registry, data set, or biobank	25 (41.0)
Advocated to government or other authorities for research funding, other research resources, or changes in regulations relating to research	22 (36.1)
Participated in a committee within a government or research body responsible for research decision-making	12 (19.7)
Provided information or counselling to assist participants in research studies	21 (34.4)
Disseminated information about research (for example, by making information about research findings available on your website)	48 (78.7)
Conducted the organisation’s own research	16 (26.2)
None of the above	3 (4.9)

^a^Totals equal more than 100 % because respondents could choose more than answer

#### Research funding

Fifty-nine per cent of the surveyed RDPOs had provided funds for research in the last five years (Table 3). Australian RDPOs generally do not provide large sums of money relative to the high costs of conducting scientific and medical studies. Except for one organisation that gave more than $1,000,000, no RDPO allocated more than $500,000 to research in the 2012–13 financial year. A chi-square test of the relationship between categories for “total budget” and “research expenditure” showed that RDPOs’ research funding was strongly related to their total budgets (*p* < 0.01). This was not a perfect relationship, however, one RDPO with an annual budget of more than $1 million allocated less than $10,000 to research in 2012–13. In contrast, two interviewed RDPO leaders said they dedicate “every cent” of their funds to research.

In accordance with previous findings that RDPOs are increasingly using their financial leverage to commission research projects favoured by patient communities rather than determined by researchers [10, 13], many of the leaders we interviewed had initiated and funded research projects with the explicit aim of encouraging researchers to study matters identified as priorities by the RDPO.

#### Non-financial support to researchers

Approximately three quarters of the RDPOs (77 %) had provided non-financial support for research in the five years prior to the survey. Answers to a follow-up question about the types of non-financial support provided revealed that thirty-four RDPOs (56 %) had helped to identify potential participants for clinical trials or studies, and 28 (46 %) had helped recruit respondents for surveys. These activities may occur in the context of studies RDPOs have funded and/or at the request of researchers.

The support provided by some RDPOs extends beyond “helper” roles to proactive facilitation of connections between researchers working in different research institutions and/or disciplines. Several study participants noted that collaboration between researchers is particularly important for rare disease research, which often requires co-operation between different institutes to ensure sufficient numbers of patients or biospecimens. Some leaders expressed strong sentiments about their organization’s role in bringing researchers together. For example:

*We formed a collaborative of international and Australian institutions…If you’re playing football, it’s a lot easier to pass the ball to someone else to get to the goal line quicker. So we’re embracing the collaborative aspects. We’re big on working with people.*

*– Paul, interviewed RDPO leader*

Other RDPO leaders encouraged connections between researchers in more subtle ways; for example, by keeping researchers informed of each other’s work or organising meetings allowing them to share information directly with each other. Overall, 14 (30 %) of the surveyed RDPOs that had provided research funding or non-financial support agreed that they “had undertaken actions aimed at creating closer links between different researchers”.

#### Patient registries and biobanks

Twenty five (41 %) of the surveyed RDPOs had contributed to a patient registry or biobank established by a research institution. The survey did not obtain details about the nature of these contributions, but website content and comments made by study participants indicated that RDPOs may support registries and biobanks financially; by advising on their design or evaluation; or by encouraging patients and families to contribute their data and/or their biospecimens (usually via a treating clinician).

In addition to the RDPOs that contributed to researchers’ registries or biobanks, seven RDPOs (11 %) had managed their own patient registries in the last five years. Internationally, RDPOs are increasingly establishing their own registries and biobanks to facilitate research on their diseases [56–58]. However, given that establishing and maintaining registries and biobanks is complex and resource intensive, this trend has led to concerns about quality assurance and ethical oversight of RDPO-managed collections of patient data and biospecimens [58, 59].

#### Research-related lobbying

The data in Table 3 show that RDPOs privilege more direct forms of involvement in research over lobbying activities. Only 36 % of RDPO leaders reporting having “advocated to government or other authorities for research funding, other research resources, or changes in regulations relating to research” in the last five years. Supporting claims by previous authors that disease-specific RDPOs prioritise direct engagements with researchers because they lack the organisational capacity and numbers required for successful lobbying of authorities [9, 60], we found that RDPOs with higher annual budgets and numbers of paid staff were significantly more likely to undertake lobbing activities (*p* < 0.05).

#### Participation in research committees

Only twenty per cent of the surveyed RDPO leaders had participated in committees established by government or research bodies responsible for research decision-making. Further highlighting the importance of RDPOs’ financial resources in terms of opportunities to influence research, we found a strongly significant association between RDPOs’ annual budget and their participation in research decision-making committees: wealthier organisations were much more like to have these opportunities (*p* < 0.01).

#### Providing information or counselling for research participants

Some Australian RDPO leaders have developed a sophisticated understanding of research governance and use this knowledge to advise members of their rights as study participants and the risks and benefits of their participation. About a third of the surveyed leaders (34.4) had “provided information or counselling to assist participants in research studies” (Table 3).

#### Disseminating research information

Disseminating research information, which had been done by 79 % of Australian RDPOs (Table 3), is an important form of engagement with research. Many RDPO websites showed that leaders had compiled information about current research projects and findings. Interviewed leaders gave examples of how this information had led organisational members to develop ideas for further research, which they then used in discussions with researchers or as a basis for awarding research funding.

#### Studies conducted by RDPOs

Sixteen (26 %) of those surveyed indicated that their RDPO had initiated and carried out their own research in the last five years. RDPOs’ research projects were usually led by volunteers associated with the organisation. However, in three cases the research was conducted by professionals employed or contracted by RDPOs.

Leaders’ explanations of why their RDPO conducted its own research revealed two main themes: first, an inability to attract professional researchers to conduct research on the disease and, second, a perceived need for more information about the psychosocial impacts of the disease. Examining RDPOs’ independent research in the context of other activities reported in the survey and on their websites, it appeared that their studies were usually conducted in the hope that their findings would influence professional researchers, clinicians or policy makers. Five of the 16 respondents made this motivation explicit in their comments.

While some commentators claim that patient organisations are increasingly conducting their own evaluations of therapies they believe could help treat their disease [61–64], we found no evidence that Australian RDPOs had organised interventional clinical studies independently of the research establishment. However, more than a third (38 %) of the surveyed leaders were aware of members of their organisation who had participated in “participant-led” clinical studies (for example, drug tests conducted by groups of people who connect online) independently of both the RDPO and biomedical researchers.

### Perceived challenges

Key challenges identified by our study participants are discussed below.

#### Lack of RDPO resources and expertise

Insufficient funding and consequent limitations of organisational capacity were the factors most frequently cited by surveyed respondents when asked to explain why their organisations had not been involved in research in the way they would wish. This was also a prominent theme in interviews with RDPO leaders.

Although the leaders who participated in the survey and interviews believed their own research funding was well managed, several suggested that “other” RDPO leaders lack the expertise needed to direct their organisation’s research funding in ways most likely to have the desired impact. This is because RDPO leaders are (usually) non-scientists, often with little experience of financial or contractual governance, who may struggle to identify research projects and funding strategies that make best use of their resources. A number of study participants expressed concerns that RDPO leaders may not obtain informed and impartial advice on the research they plan to fund. Although about 80 % of the “research funder” RDPOs indicated they have scientific or medical advisors, these same advisors may be potential recipients of the RDPO’s funds. The difficulty of obtaining impartial expert advice was noted by a survey respondent:

*It is very important to seek independent advice from one's SAC [scientific advisory committee] and to have a SAC comprising of experts of the best disease reputation and integrity. Finding world renowned advisors in whom you have faith would recommend the best project [even if it was] competing against one from their own lab is harder than you think and it takes a lot of critical information and knowledge and networking to acquire this confidence.*

*– Survey respondent*

#### The research environment

Comments about RDPOs’ limited resources and expertise comprised about two thirds of survey respondents’ answers to the open-ended question on factors preventing them from being involved in research in the way they would wish. However, a third of the comments referenced aspects of the external environment. Several respondents claimed that there were no or few Australian researchers “interested” in their diseases – although it was not clear if they attributed this to lack of funding or researchers’ unwillingness to study rare diseases. Other respondents commented explicitly that rare diseases are neglected in Australian government research funding allocations, relative to their prevalence in the population and the impact on individuals and society.^5^ A few attributed this to the government’s strong focus on Australia’s national health priorities, which are common diseases [65]. Several respondents also commented that research on their diseases was impeded by the lack of a mechanism for collecting patients’ demographic and clinical data: these respondents called for the development of an Australian rare disease patient registry, as exists in the United States [56] and many European countries [66].

While survey respondents were, overall, more satisfied than dissatisfied with the “extent to which researchers listen to advice and input from the RDPO” (their average rating of 3.6 out of 5 indicated a position somewhere between “neither satisfied nor dissatisfied” and “satisfied”), several respondents stated that attitudes and practices within the research community – specifically, researchers unwillingness to work collaboratively with patient organisations or take account of their ideas – was a barrier to the RDPO’s involvement. For example:

*Walking the walk is not our problem but being volunteers is perceived by some as incongruous with having skill, expertise and deep knowledge.*

*– Survey respondent*

*There is no collaboration between our organisation and the researchers about possible opportunities for patient family members to contribute or requests for involvement apart from the provision of funding.*

*– Survey respondent*

However, while some participants in our study were critical of researchers’ perceived reluctance to involve RDPOs in their work, others recognised the challenges of incorporating lay perspectives into scientific studies. According to a key informant, the effectiveness of involving RDPOs in research decision-making and deliberative processes may be limited by representatives’ lack of preparation for these roles:

*I know from the projects that we’re involved in…there’s a big emphasis on having patients involved in the planning and the setup of these research projects. But the extent to which they have the knowledge and the training that might be necessary for them to make an informed [contribution]…I mean that sounds paternalistic but, you know, to be able to contribute in a way that’s in their best interest, I’m not sure how much training they’re getting prior to being asked to participate as a representative for a patient organisation.*

*– Melanie, key informant*

## Discussion

Despite differences in their respective research environments, comparison of our survey data with available data from Europe and the US suggests that Australian RDPOs are similar to their international counterparts and engage in relationships with researchers in broadly the same ways. Importantly, surveys in Europe and the US, respectively, show that most RDPOs in those jurisdictions are also small, low-budget organisations led by volunteers [35, 67].

There are, however, some specific differences between our findings and results of a survey of European RDPOs conducted by EURORDIS in 2009 [34, 67]. First, the 59 % of Australian RDPOs that had provided funds for research in the five years before our survey is notably higher than the 37 % of European RDPOs reported to have funded research in the five years prior to the EURORDIS survey [34]. Second, while only 20 % of our surveyed leaders agreed that they had “participated in a committee within a government or research body responsible for research decision-making”, 30 % of European RDPO leaders answered affirmatively when asked a more limited survey question about “participation in scientific committees within institutions” [67]. Third, compared with the 49 % of European RDPOs reported to have provided information for “clinical trial participants” in the five years prior to the EURORDIS survey, only 34 % of Australian RDPOs had provided information for participants in any form of research.

These differences may reflect better funding of rare disease research and greater opportunities for patient participation in deliberative processes in Europe, and the influence of organisations such as EURORDIS in helping RDPOs to support research participants and patient advocates. European RDPO leaders may feel greater confidence that research will progress without their financial assistance and may channel their desire to support research in other ways. Noting that our survey was conducted four years after the EURORDIS survey, recent developments further supporting European rare disease research [68] and RDPOs’ research involvement [23] may have accentuated the differences we observed.

### Contributions and constraints associated with RDPOs’ research involvement

Compared with previous surveys, our study’s inclusion of in-depth interviews and website review allowed for a richer understanding of the experiences and views of RDPO leaders. Given that most RDPO leaders in our study reported significant challenges in meeting their research aspirations – which they usually attributed to the organisation’s small size and resources – we believe that the focus in academic and popular literature on RDPO “success stories” may have diverted attention from the struggles and challenges experienced by the majority of RDPOs when trying to influence research. We contend that the types of organisations highlighted in the literature – such as those led by highly driven individuals with significant business and entrepreneurial skills [10, 15, 21, 69]; large professionalised organisations with significant financial clout [70–72]; and political activists [73] – constitute only a minority of RDPOs.

Consistent with popular narratives of RDPOs’ impact on research, a few Australian RDPOs have helped achieve significant scientific and therapeutic advances. For example, Muscular Dystrophy Western Australia is recognised as a major financial contributor to ground-breaking therapy development conducted at the Australian Neuromuscular Research Institute [74]. The leader of another Australian RDPO, Mission Massimo [75], has spearheaded important discoveries through initially conducting desktop research and then recruiting scientists to further investigate his ideas.

While in most cases the outcomes to date of RDPOs’ involvement are less dramatic, it is clear that patient organisations contribute in a variety of ways to rare disease research, and not merely through fundraising. By establishing networks of patients and their families nationally or even internationally, RDPOs have contact with dozens or even hundreds of individuals affected by a rare disease and they can therefore help researchers overcome the difficulty and high cost of accessing study participants, patient data and biological specimens. RDPO leaders may propose novel ideas for research, based on the experiential expertise of patients and families, their reading of relevant biomedical literature and – in some cases – research conducted by the RDPO itself. Many leaders also act as research “intermediaries” [76], taking an active role in forging connections between researchers in different areas and institutions. Such connections are increasingly recognised as being critical to the future of rare disease research and biomedicine generally, particularly the translation of scientific discoveries to clinical therapies [77–79].

As well as contributing funding, logistic support and expertise – which may facilitate scientific advances – a second important role of RDPOs is to help align the work of researchers and policy-makers with the needs of the public. This is an explicit aim of the current Australian Government [80]. Similar to many other developed nations [81–83], the Government has policies encouraging publicly funded researchers to involve patient/consumer representatives in their work [84, 85]. Such policies are based on the belief that there is ethical value in ensuring patients’ voices are heard [11] and that their participation will provide “real world” information about health conditions and the impacts of research practices on patient communities [86, 87]. Despite the existence of “consumer involvement” policies, however, only a minority of Australian RDPOs have the opportunity to be involved in the formal decision-making processes of researchers and policy-makers.

A third way in which RDPOs contribute to research is by empowering research participants and thus helping to uphold established standards of ethical research conduct. As trusted sources of information for people affected by rare diseases, RDPOs can help to protect and support patients and family members who take part in research studies. Not only do some leaders educate their members about their rights and the potential risks and benefits of their participation in studies, but they may also provide information aimed at helping patients and families to be more sophisticated consumers of information provided by researchers, the media, and marketers of therapies. While responsibility for research integrity ultimately rests with researchers and policy-makers, the role played by patient organisations is recognised internationally by bioethicists [86, 88].

Despite the value of their involvement in research, however, RDPO leaders identify considerable challenges associated with their research goals. As mentioned, leaders themselves explain these challenges mainly in terms of lack of resources, but we believe two underlying factors may contribute to their difficulties: a growing number of small, uncoordinated RDPOs and lack of policy support in Australia for rare disease research and patient advocacy.

### RDPO proliferation and fragmentation

In accordance with evidence from the US that the number of disease-specific non-profit organisations has been rising sharply since the 1990s [89], it appears that new RDPOs are being formed at a greater rate than in the past. A key informant to our study said that this growth has been driven by the internet (which enables RDPOs to form around even extremely low prevalence diseases) and new knowledge about the genetic underpinnings of diseases (which has resulted in some RDPOs splintering into groups devoted to specific forms of related diseases). These observations are supported by our survey data showing that RDPOs formed in the last decade focus on lower prevalence rare diseases and have lower membership numbers than older groups. Given our survey data showing that RDPOs’ opportunities to be involved in research (either financially or in other ways) are related to their size and resources, it is likely many of these smaller RDPOs will struggle to achieve their research goals. This was suggested by an interview participant.

*My experience is that there are many* [RDPOs] *that overlap, that do the same thing: you’ve got quadrupling or tenfold…of administrative costs. Running a non-profit is not a trivial exercise…the workload is immense…If you’re going to have all these fundraising dinners to feel good about it, and I hate to sound harsh in saying this, but if you’re going to raise a thousand dollars at the end of twelve months effort that’s taken ten thousand hours of work, you’re better off to go get an extra job, make a thousand dollars and donate it to another charity.*

*– David, interviewed RDPO leader*

As well as reducing the ability of individual RDPOs to attract funds from affected individuals and the public, the growth and fragmentation of RDPOs may mean that they neglect opportunities for collaboration. While we found little evidence that different RDPOs had collaborated for research purposes, it has been suggested that joint studies funded by patient organisations working in the same or similar disease areas (for example, different diseases causing epilepsy or different blood disorders) would provide economies of scale and might also identify common disease pathways and drug targets [4]. RDPOs’ focus on their own specific diseases may also compromise their involvement in collaborative efforts to influence government policy on matters of common concern – such as funding for rare disease research infrastructure and regulations relating to genetic testing, and therapy development, approval and subsidisation.

#### Lack of supporting policies and programs in Australia

Australian RDPOs currently rely mainly on their own expertise and resources when trying to advance research, as there is no policy framework to help them develop mutually beneficial relationships with researchers. In contrast, European patient organisations are actively engaged in RD-Connect, a EU-funded initiative linking rare disease registries, biobanks and other databases [28] and in the development of outcome measures for clinical trials, therapy approval, and post-marketing drug surveillance [23, 49]. In the US, each of the research consortia in the National Institutes of Health’s Rare Diseases Clinical Research Network program directly involves patient organisations as well as researchers – enabling scientists, clinicians and people affected by rare diseases to work together on disease-specific projects [50]. US examples also include the Patient-Centred Outcomes Research Institute [90]: this is not specific to rare diseases, but the Institute’s Advisory Panel on Rare Diseases provides guidance on how to foster collaborative approaches between researchers and RDPOs [14].

For RDPOs without the capacity to raise substantial funds for research, opportunities to be involved in research depend on the level of research activity funded by government and industry. In Australia, the ability of RDPOs to contribute to research is therefore constrained by the fact that the research environment provides few incentives for researchers to study rare diseases (see Background section).

### Opportunities to strengthen RDPOs’ involvement in research

Our study was not designed to develop or evaluate policies: however, its findings do highlight opportunities to strengthen RDPOs’ involvement in research, and enhance the value of this involvement for both researchers and RDPOs. We outline some preliminary suggestions below for further investigation and discussion with stakeholders.

#### National leadership in rare disease policy and planning

There has been significant government leadership in the development of rare diseases national plans in other jurisdictions, particularly in Europe [25]. The plans provide a framework for coordination and monitoring of the scarce resources associated with various rare diseases, with the aim of creating new opportunities for research, service provision and patient group engagement. An Australian National Rare Disease Plan could address some of the issues highlighted by our findings, such as lack of data on rare disease research projects and funding; fragmentation of resources for rare disease research; lack of opportunities for patient organisations to be involved in research unless they have significant funds; and a perceived lack of fairness in the allocation of Government research funding.

To date, however, calls by the Western Australian State Government [38, 40], researchers [39, 91], medical professionals [92] and an Australian rare disease advocacy group [52] for a nationally coordinated approach to rare disease planning have gone unheeded. We recommend that the Australian Government should work with rare disease advocates, researchers and other stakeholders towards the development and implementation of a national plan for rare diseases.

#### Support for collaboration between researchers and patient organisations

As the main Australian Government agency responsible for biomedical research policy and funding, the National Health and Medical Research Council (NHMRC) would be well placed to take the lead in connecting patient organisations and researchers, and developing resources to help them work together. A participant in our study suggested that NHMRC could extend its existing initiative, negotiated by a small group of RDPOs, in which RDPOs are advised of relevant, high-quality research applications that the NHMRC is unable to fund. By arrangement with the NHMRC, RDPOs could also ask researchers applying for their funding to first submit their proposals to the NHMRC. This would enable patient organisations to access expert, independent advice and to direct their funds to studies that have scientific merit and value for the patient community.

#### Infrastructure for rare disease research

As suggested by some of our study participants, the environment for rare disease research in Australia would be greatly improved by a nationally coordinated approach to rare disease research infrastructure, in particular through establishment of patient registries and biobanks. Well-recognised problems relating to rare disease registry in Australia include duplication of effort and poor economies of scale in disease-specific registries; lack of consistent standards and comparability of information across different registries; a lack of registries for many rare disease communities; the short-term nature of registries created for specific research projects; and perceived inadequacies in patient/parent input to the design of some registries [93]. Similar to registries, biobanks for rare diseases are fragmented and lacking either inter-operability or consistent standards for specimen collection, storage, obtaining patient/parent consent, or sharing of samples between researchers [46].

An internet tool developed by Western Australian researchers presents a specific opportunity to develop an Australian rare disease registry [93]. Importantly, this registry architecture is open source – meaning that it is freely available for use or modification – and allows customised data input by patients or their family members. Therefore it could provide a secure, presumably low-cost, alternative to RDPO-managed registries and a basis for collaborative data collection by researchers and patient organisations. These features of the registry may lead to greater levels of data capture, which is critical in advancing knowledge about rare diseases. Further, a national registry would help the Australian research community to align itself with efforts in Europe and the US to “internationalise” rare disease research by fostering research collaborations [94] and connectivity between national and trans-national rare disease registries and biobanks [95].

#### RDPO coordination and support

Better coordination of rare disease patient advocacy and patient groups in Australia was described as “long overdue” in 2006 [92], but it was only in 2012 that a small national advocacy group, Rare Voices Australia, was formed. Rare Voices Australia, which currently relies mainly on funding from pharmaceutical companies, has focused on consultation and lobbying on issues of relevance to rare diseases, particularly the development of a national plan and patients’ access to high-cost therapies [52]. However, some resources for RDPOs wishing to engage in research are available from peak agencies for “genetic” and rare disease groups in three states (Victoria, New South Wales and Western Australia), which were established by state government health departments in the 1980s and 1990s.

In our view, an important aspect of the proposed National Rare Disease Plan would be to articulate the respective roles of Australia’s national and state-based rare disease alliances. These organisations should work together to ensure optimal use of their collective resources. Our study findings point to several ways in which rare disease alliances could support the research aspirations and activities of disease-specific patient organisations: first, by providing guidance for RDPOs that fund or otherwise engage in research; second, by engaging with RDPOs in order to reflect all points of view in research-related advocacy; third, by helping RDPOs to safeguard the welfare of their members who participate as “subjects” in traditional and “participant-led” research; and, fourth, by promoting collaboration between RDPOs, including collaboration on joint research initiatives. The Australian Government should give consideration to appropriate funding models and levels to enable the national and state-based alliances to carry out these roles without undue reliance on the pharmaceutical industry.

## Conclusions

In this article, we have explored the research-related goals, activities and challenges of a sizable group of Australian RDPO leaders. Although a number of other countries have better-developed policies and programs supporting RDPOs’ involvement in research, many of the issues we identify in this Australian sample of RDPOs are likely to be applicable to RDPOs in other parts of the developed world.

In the context of scarce government and industry funding for rare disease research, the funds and logistical assistance provided by RDPOs may significantly advance research. Beyond their material and practical contributions, RDPO leaders and their members possess knowledge that can enrich research and better align its directions and practices with the needs of people affected by rare diseases. Despite the value of their involvement, however, RDPOs face significant challenges in achieving their research goals. Most leaders focus on fund-raising, expertise and resourcing issues when explaining their difficulties, but it is clear that their problems are exacerbated by the fragmentation of the RDPO sector and the marginalisation of rare diseases in health and research policy.

We have suggested that there are considerable opportunities in Australia, building on the findings of this study and work conducted internationally, to strengthen and safeguard RDPOs’ involvement in research. Such initiatives – ideally implemented as part of a nationally coordinated and government-supported plan to improve rare disease research, services and patient advocacy in Australia – could help advance scientific knowledge and therapy development, thereby alleviating the personal and societal burdens of rare diseases.

## Abbreviations

EU: European Union
EURORDIS: European Organisation for Rare Diseases
NHMRC: National Health and Medical Research Council
RDPO: Rare disease patient organisation
US: United States

## Footnotes

1 While the Australian Therapeutic Goods Authority considers a rare disease to be a condition, syndrome or disorder that affects 1 in 10,000 people or less, there is a strong push from Australian rare disease advocates for a nationally consistent definition aligned with the European Union (EU) definition, which refers to an estimated prevalence of less than 5 in 10,000 (1 in 2000). Our study uses the EU definition.

2 Because six survey respondents chose not to identify themselves, there was a small chance that an RDPO classified as a “non-responder” had in fact participated in the survey.

3 Estimate based on website information. The survey asked about the number of paid staff, but did not distinguish between paid leaders and paid staff performing other roles (for example, such administration or fundraising). The estimate includes survey respondents from separate state branches of large federated RDPOs, with paid leaders in each state as well as a paid national leader.

4 Including the four respondents who had no research or research advocacy goals, and who were therefore not asked the question.

5 It is not possible to check the accuracy of participants’ perception that rare diseases as a group are under-funded in Australian research expenditure, as the Government’s main research funding body, the National Health and Medical Research Council, does not provide data on allocations to rare disease research.
